# Socio-demographic Profile of Clinical Medico-legal Gender-based Violence Cases at Jumla Nepal: An Observational Study

**DOI:** 10.31729/jnma.v64i293.9289

**Published:** 2026-01-31

**Authors:** Apurba Acharya, Umesh Raj Aryal, Kushal Bhattarai, Jeetendra Bhandari, Sushma Kaphle, Ashna Parajuli, Arbin Shakya, Nilu Manandhar

**Affiliations:** 1 Institute of International Health, Charite University of Medicine, Berlin, Germany; 2 Biostatistics and Research, Karnali Academy of Health Sciences, Jumla, Karnali Province, Nepal; 3 Department of Clinical Biochemistry, Rapti Academy of Health Sciences, Dang, Lumbini Province, Nepal; 4 Department of General Practice and Emergency Medicine, Karnali Academy of Health Sciences, Jumla, Nepal; 5 Department of Clinical Pharmacology, Karnali Academy of Health Sciences, Jumla, Karnali Province, Nepal; 6 Global Health Research Center for Multiple Long Term Conditions, Kathmandu Medical College, Bagmati Province, Nepal; 7 Department of Forensic Medicine, Bheri Hospital, Banke, Lumbini Province, Nepal; 8 Department of Clinical Physiology and Biophysics, Rapti Academy of Health Sciences, Dang, Lumbini Province, Nepal

**Keywords:** *assault*, *clinical forensics*, *gender-based violence*, *rural Nepal*

## Abstract

**Introduction:**

Gender-based violence is a burden throughout Nepal. This study aimed to assess the sociodemographic profile of Gender-based violence cases and shed light on factors associated with these incidents in the Jumla district of rural Nepal.

**Methods:**

A retrospective observational study involving all the Gender-based violence cases brought for clinical medico-legal examination at the Department of Forensic Medicine and Toxicology, Karnali Academy of Health Sciences, Jumla, Nepal, from January 01, 2022 - December 31, 2023. The study variables included various socio-demographic profiles of these cases. These variables were entered and analyzed using Microsoft Excel (version 16.16.27--201012) and SPSS 23.0.

**Results:**

Among the total clinical forensic medicine cases, Gender-based violence was present in 83 (41.92%). All the females were survivors, 53 (63.85%), and all the malex were perpetrators, 30 (36.14%). The median ages of the female and male were 20 years (Range: 11-73) and 19 years (Range: 13-52), respectively. Among these cases, 20 (24.10%) had physical assault, and 63 (75.90%) were related to sexual assault, with all male examinees presenting with sexual assault.

**Conclusions:**

Gender-based violence is common among females in Jumla and is primarily inflicted by males due to various sociocultural factors.

## INTRODUCTION

Gender-based violence (GBV) is one of the major burdensome public health concerns. It can be any form of violence directed against any individual on the basis of their gender, irrespective of their age, sex, or socioeconomic status.^1^ Although sexual violence is the major entity of GBV, it also includes domestic violence, psychological and emotional harassment, intimate partner violence, sex trafficking, early marriage, financial abuse, and structural violence from gender-based traditions.^2,3^

In Nepal, almost half of women (48%) have experienced different forms of violence during their lifetimes.^2^ In the present study site, a report circulated by the district police office revealed a sustained increase in violence cases in the last three fiscal years, suggesting a pattern of increased GBV.^4^

The primary objective of this study is to assess the sociodemographic profile of individuals exposed to GBV and explore the relationship between the survivor and the perpetrator.

## METHODS

This retrospective observational study was conducted at Karnali Academy of Health Sciences (KAHS), Chandannath, Jumla, Karnali Province. The center with forensic medicine specialists receives cases of clinical forensic medicine for medico-legal examination at its Department of Forensic Medicine and Toxicology (DoFMT). These cases predominantly include physical assault and sexual assault, with occasional age-estimations. In the present study, ethical clearance was obtained from the Institutional Review Committee of KAHS (ref. no. 080/081/34). A purposive sampling method was used including all gender-based violence (GBV) cases that were brought for clinical medico-legal examination at KAHS within the two years of the study period from 1^st^ January 2022-31^st^ December 2023. Whether the cases were associated with GBV or not was distinguished from the history provided by the examinee. The study excluded all clinical forensic medicine cases that were not associated with GBV. The study also excluded GBVs which did not seek for clinical medico-legal examination and were managed by the hospital-based One-stop crisis management center (OCMC).

The data were collected from the register of clinical forensic medicine, and the details of the history provided by the examinee were obtained from the official copy of the dispatched clinical forensic medicine reports, both of which are maintained within the DoFMT. These two documents were used for secondary data, and variables such as age, sex, marital status, municipal level, site of incident of GBV, relationship with the examinee, type of assault, and associated injuries were obtained.

The age of the examinee was further categorized as less than 13 years, 13-19 years, 20-29 years, 30-39 years, 40-49 years, 50-59 years, or 60 years or above. Marital status was categorized as married or unmarried. The municipal level of the examinee was documented on the basis of the eight municipal levels of the Jumla district, namely, Chandannath, Guthichaur, Tatopani, Patarasi, Tila, Hima, Sinja, and Kanakasundari. The site of the incident was categorized on the basis of its occurrence at home, within the same municipal level, and within a different municipal level. The relationships of the examinee with the alleged perpetrator or the survivor were classified as family members, which included the father, mother, son, daughter, husband or wife of the examinee; relatives, which referred to all the relationships, excluding those in family; neighbours; strangers, referring to those whom the examinee did not know prior to the incident; and others, referring to those who were allegedly boyfriends or girlfriends of the examinee and those with whom the examinee had at least prior telephone conversations. Furthermore, the type of assault was recorded as physical assault or sexual assault. The associated physical injuries were documented as blunt force injuries, sharp force injuries or no injuries.

Data were entered and analyzed using Microsoft Excel version 16.16.27 (201012) and IBM SPSS Statistics version 23. Categorical variables were summarized using frequencies and percentages to describe the distribution of key characteristics among the study participants. Continuous variables were analyzed by calculating the median and range. Graphical representation, specifically bar diagrams, was used to illustrate the relationships between the examinees and the perpetrators or survivors.

## RESULTS

During the two-year study period, a total of 198 clinical forensic medicine cases were presented to the DoFMT for medicolegal examinations. Based on the brief history provided in the dispatched reports of all examinees associated with GBV, the documented survivors were exclusively female, and the alleged perpetrators were male. Among all the clinical forensic medicine cases, 83 (41.92%) were of GBV, with 53 (63.85%) presented for examination as a survivor and 30 (36.15%) as a perpetrator. Among these GBV cases, 20 (24.10%) had physical assault, and 63 (75.90%) were related to sexual assault. The median ages of the female and male were 20 years (Range: 11-73) and 19 years (Range: 13-52), respectively (Table 1).

**Table 1:** Sociodemographic characteristics of female examinees associated with GBV (n=53).

Characteristics	n(%)
Age in Years	
<13	2(3.77)
13-19	23(43.39)
20-29	13(24.52)
30-39	4(7.54)
40-49	5(9.43)
50-59	3(5.66)
60 and above	3(5.66)
Marital Status	
Married	27(50.95)
Unmarried	26(49.05)
Municipal Level of Examinee	
Chandannath	18(33.96)
Tatopani	3(5.66)
Guthichaur	13(24.52)
Patarasi	2(3.77)
Tila	8(15.09)
Hima	6(11.32)
Sinja	2(3.77)
Kanakasundari	1(1.88)

**Figure 1:**
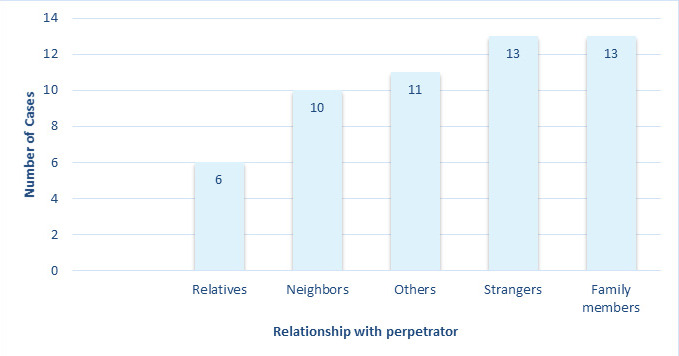
Relationship of the female examinee with the alleged perpetrators (n=53).

Further, the relationship of the female examinee with the alleged perpetrators have been shown in Figure 1.

Among all the female examinees who were exposed to GBV, the site of the incident at home or within the same municipal level was 21 (39.62%) each, 11 (20.76%) incidents occurred at a different municipal level other than their homes. Additionally, 33 (62.26%) of the GBV cases were due to sexual assault, and 20 (37.74%) were due to physical assault. Furthermore, across all GBV cases, 32 (60.38%) were not associated with any physical injuries, and 21 (39.62%) had blunt injuries with 2 (9.52%) and 19 (90.48%) female examinees sustaining sexual and physical assault, respectively.

Among all the male examinees who were allegedly perpetrators, a total of 22 (73.33%) incidents of GBV were within the same municipal level, followed by 7 (23.34%) incidents at home, and only 1 (3.33%) incident occurred at a different municipal level than their homes. All the male examinees were presented with a history of sexual assault. Furthermore, across all the male examinees, 29 (96.67%) were not associated with any physical injuries, and only 1 (3.33%) had blunt injuries related to sexual assault (Table 2).

**Table 2:** Sociodemographic characteristics of male examinees associated with GBV (n=30).

Characteristics	n(%)
Age in years	
Less than 13	1(3.33)
13-19	15(50.00)
20-29	6(20.00)
30-39	4(13.33)
40-49	2(6.67)
50-59	2(6.67)
Municipal Level of Examinee	
Chandannath	6(20.00)
Tatopani	4(13.33)
Guthichaur	7(23.33)
Patarasi	4(13.33)
Tila	6(20.00)
Hima	3(10.00)
Marital Status	
Married	16(53.33)
Unmarried	14(46.67)

**Figure 2:**
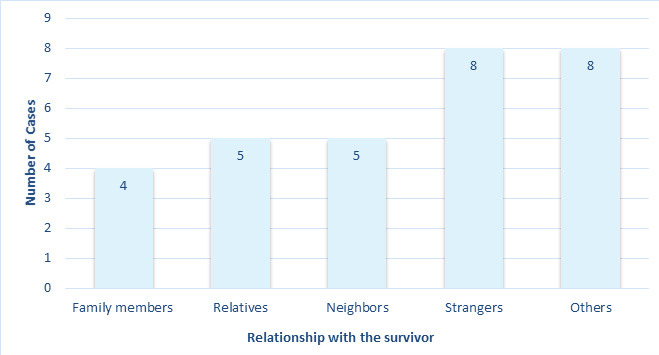
The relationship of the male examinee with the alleged survivors (n=30).

Further, the relationship of the male examinee with the alleged perpetrators have been illustrated in Figure 2.

## DISCUSSION

GBV is a glaring concern globally and is deeply rooted across cities and communities of rural and urban Nepal. Jumla district is one such region of the country in midwestern Nepal, where conventional sociocultural views have persisted through generations and have led to multiple generations of gender discrimination and inequality, which has contributed to the persistence of GBV.^5,6^ The results of the present study emphasize that almost half of the medico-legal cases seeking clinical forensic medicine services were associated with GBV.

A study by Dangal et al. in Dolakha district, central Nepal, reported that 93.7% of the participants in all the cases of GBV seeking either medico-legal services or only medical services were female, and 7.3% were male. Compared with the present study, there were no male cases of GBV in Jumla who sought clinical medico-legal services.^3^ This difference across the two studies could be due to the utilisation of all the cases received by the hospital over a 5-year study period by Dangal et al.^3^ This difference could also be partly due to the presence of an ongoing COVID-19 pandemic during the study period of Dangal et al. when there was a global upsurge in GBV cases, as reported by Akudolu et al and Phillimore et al.^3,7,8^ According to the brief histories recorded in the dispatched reports of all GBV-related examinees, the reported survivors were all female, while the alleged perpetrators were all male. However, this pattern should be interpreted with caution, as it may reflect underlying reporting biases and cultural norms that influence disclosure. In many settings, male survivors and female perpetrators may be underrepresented due to stigma, societal expectations, and reluctance to report such cases. A study incorporating districts of eastern Nepal by Yadav et al. reported that all the cases of sexual assault were associated with female survivors, with males being predominantly associated with physical assault.^9^ This high number of female survivors associated with sexual assault was similar to that reported in another study conducted by Baral et al. in the Kaski district, western Nepal.^10^ However, unlike the present study, which included the maximum number of cases across the 13-19 years age group, the study by Baral et al. included the maximum number of cases across the 20-30 years age group.^10^ Additionally, none of the cases of male perpetrators of GBV were associated with physical assault in the present study.

In terms of marital status, almost equal numbers of survivors and perpetrators in the present study were either married or unmarried. For all the female survivors, nearly half the incidents of violence were inflicted by family members or strangers at home or within the same municipal level as their home. However, for all the male perpetrators, the survivors were either strangers or those categorized as others, with the incidents of violence occurring mostly within the same municipal level of the perpetrator’s home. The distribution of cases, similar to those in the present study, of the survivors, irrespective of marital status, and those who inflicted violence, was also highlighted in a study by the Government of Nepal in 2012.^11^ The findings provided by the government of Nepal over a decade ago still holds relevance in Nepalese society, with a significant number of female survivors being afflicted by violence within their own households.

GBV refers to any form of violence to an individual regardless of their gender. However, in the present study, the distribution of cases revealed a glaring pattern in which male perpetrators were associated with GBV irrespective of the type of violence, place of violence, and marital status across all age groups. This pattern, with high male preponderance, is not just visible in the present study but is equally apparent in the studies across different parts of the country.^3,9,10,12^ This increased number of male-led GBV cases may be due to the perpetuation of traditional gender-specific roles, which are still a predominant part of our societal establishments and an ever-prevalent patriarchal norm and supremacy. This, combined with limited opportunities for education and employment in rural parts of Nepal, such as Jumla, has created a lack of autonomy, minimal awareness in response to the threats of violence, limited communication, and ever-growing financial dependence, all of which lead to the risk of violence and vulnerability to females.^11,12,13^

The highest number of survivors in the present study arrived from Chandannath municipality. This could be due to the institution being within the same municipality. The geographical terrain of Jumla across all eight municipal levels is extreme, with temperatures as low as -14 degrees Celsius during winters and challenging transportation routes.^14^ As such, survivors traveling from within the same district may sometimes require over a day to reach institution-seeking services. Additionally, the financial burden of traveling for these survivors and the social stigma associated with sharing their journey through violence minimizes their desire to seek medico-legal or psychosocial services. Additionally, the awareness campaigns on GBV throughout these municipal levels are curtailed due to the difficult terrain of Jumla.

One of the major concerns with GBV is the possible consequences associated with it. GBV can occur in various forms and can present health-related outcomes both physically and psychologically. In the present study, across both male and female examinees, only 22 (26%) of the cases were associated with physical injuries. However, the psychological effects of these factors were not further evaluated in this study. These violent events in rural communities place a significant burden on survivors’ well-being for the rest of their lives, as these events are intricately interconnected within society and their families and can pose serious consequences in the life course, having an intergenerational impact.^15^

This study is subject to several important limitations. Primarily, it only includes cases of GBV that were formally reported and brought to the forensic department, thereby excluding a potentially large number of survivors who, due to fear, stigma, or lack of access to services, do not disclose their experiences or seek medico-legal assistance. This underreporting introduces a significant bias and limits the generalizability of the findings to the broader community. The study also only includes physical and sexual components of GBV, excluding the emotional, psychosocial and financial components. Additionally, the data reflected only female survivors and male perpetrators, which may not accurately represent the full spectrum of GBV dynamics. The absence of male survivors and female perpetrators in the study likely results from cultural norms, social expectations, and potential sampling limitations. These factors underscore the need for cautious interpretation and highlight the necessity for broader, more inclusive data collection methods in future research.

This study highlights the need to broaden GBV research which can help uncover cases that often go unreported. Preventive efforts should focus on raising awareness, teaching respect and equality from an early age, ensuring fair access to resources, strengthening legal protection including psychosocial support for the survivors, and ensuring accountability for offenders. Community engagement, primarily through culturally appropriate outreach involving local leaders, is also vital. Together, these measures can build a more effective, inclusive, and sustainable response to GBV in the region.

## CONCLUSION

GBV is widespread among young adults across nearly all municipal areas of Jumla, taking the form of both sexual and physical assault. Socioeconomic and cultural factors appear to play a significant role in the high prevalence of male-perpetrated cases.

## Data Availability

The data are available from the corresponding author upon reasonable request.
